# Draft Genome Sequence of Halobacillus trueperi SS1, Isolated from Lunsu, a Saltwater Body in the Northwest Himalayas

**DOI:** 10.1128/MRA.01710-18

**Published:** 2019-03-07

**Authors:** Sonika Gupta, Parul Sharma, Kamal Dev, David J. Baumler, Anuradha Sourirajan

**Affiliations:** a Faculty of Biotechnology, Shoolini University of Biotechnology and Management Sciences, Solan, Himachal Pradesh, India; b Department of Food Science and Nutrition, University of Minnesota Twin Cities St. Paul, Minneapolis-St. Paul, Minnesota, USA; c Microbial and Plant Genomic Institute, University of Minnesota Twin Cities St. Paul, Minneapolis-St. Paul, Minnesota, USA; d Biotechnology Institute, University of Minnesota Twin Cities St. Paul, Minneapolis-St. Paul, Minnesota, USA; Loyola University Chicago

## Abstract

We report here the genome sequence of halophilic Halobacillus trueperi SS1, isolated from the Lunsu saltwater body in India. The bacteria are Gram positive and rod shaped. The genome of H. trueperi SS1 has 4.14 Mbp, with 4,329 coding sequences, 35 RNA genes (29 tRNAs, 2 rRNAs, and 4 noncoding RNAs), and 42.15% G+C content.

## Abstract (precis)

We report here the genome sequence of halophilic Halobacillus trueperi SS1, isolated from the Lunsu saltwater body in India. The bacteria are Gram positive and rod shaped.

## ANNOUNCEMENT

Saltwater lakes and salt mines are found across the Himalayas, yet their unique flora and fauna largely remain unexplored. Halobacillus trueperi SS1 (16S rRNA gene sequence submitted under GenBank accession no. KM260166) was isolated from the soil sediments of Lunsu, a saltwater body located in Himachal Pradesh in the foothills of the northwestern Himalayas (1). Halobacillus trueperi SS1 is a strict halophile requiring at least 3.8% NaCl for growth, exhibits optimum growth at 11.6% NaCl, and tolerates up to 26.1% NaCl (1). It forms yellow-orange-pigmented colonies and produces an array of halozymes (1, 2). Despite the widespread reports of several halophiles, the mechanisms of salt tolerance have not been completely elucidated in all known halophiles. H. trueperi DSM10404 has been reported to accumulate glycine, betaine, and glutamate as compatible solutes for salt tolerance (3). We reported for the first time that H. trueperi SS1 utilizes a combination of a salt-in strategy and compatible solutes like proline, glycine betaine, and glutamate for survival under hypersaline conditions (4). To explore the salt-inducible regulons and biotechnological potential of H. trueperi SS1 (2, 4), we sequenced the entire genome of H. trueperi SS1. The H. trueperi SS1 bacterial strain was cultured in nutrient broth (NB) medium to an *A*_600_ of ∼1.0 under optimal growth conditions (1), and the cells were harvested by centrifugation at 12,000 × g for 5 min. Genomic DNA from the bacterial cell pellet was isolated as described by Sambrook et al. (5) and analyzed by agarose gel electrophoresis. The genomic DNA (200 ng) was used to prepare the paired-end sequencing library with the Illumina TruSeq Nano DNA high-throughput (HT) library preparation kit. The PCR-amplified library was analyzed in a Bioanalyzer 2100 (Agilent Technologies) using the high-sensitivity (HS) DNA chip according to the manufacturer’s instructions and loaded onto the Illumina NextSeq 500 platform for cluster generation and sequencing. A total of 1,725,613 paired-end (PE) reads with 517,683,900 bp were produced from the sequencing run. A total of 1,725,613 paired-end (PE) reads with 517,683,900 bp were produced from the sequencing run. The *de novo* genome assembly of high-quality (phred score ≥30) PE reads and scaffolding were accomplished using SOAP*denovo* version 2 (6), with a genome coverage of 130.0×. The assembled genome sequence of H. trueperi SS1 yielded 4,258,559 bp in the form of 113 scaffolds. The G+C content was found to be 42.15%. The coding sequences (CDS), RNA, and repeat regions were predicted using the National Center for Biological Information (NCBI) Prokaryotic Genome Annotation Pipeline and best-placed reference protein set of the GeneMarkS+ annotation software (version 4.6), as described previously (7, 8). A total of 4,364 CDS and 35 RNA genes (29 tRNAs, 2 rRNAs, and 4 noncoding RNAs [ncRNAs]) were predicted. One dinucleotide [(TA)_6_] simple sequence repeat (SSR) was also identified using the MIcroSAtellite (MISA) identification tool, as described previously (9). The genome annotations of the H. trueperi SS1 genome provided by the NCBI are summarized in Table 1.

**TABLE 1:** Global statistics of *Halobacillus trueperi* SS1 genome

Statistic	Annotation data
Total sequence length (bp)	4,258,559
No. of total genes	4,364
No. of total CDS	4,329
No. of coding genes	3,925
No. of coding CDS	3,925
No. of RNA genes	35
No. of rRNAs	2 (5S)
No. of complete rRNAs	1 (5S)
No. of partial rRNAs	1 (5S)
No. of tRNAs	29
No. of ncRNAs	4
No. of scaffolds	113
Scaffold *N*_50_ (bp)	81,490
Scaffold *L*_50_ (bp)	18
No. of contigs	1,533
Contig *N*_50_ (bp)	6,527
Contig *L*_50_ (bp)	203

### Data availability.

This whole-genome shotgun project has been deposited in DDBJ/ENA/GenBank under the accession no. QTLC00000000. Raw sequence reads are available under SRA accession no. SRR8351973.
